# Supplementary figures and images for: Integration of pan-cancer multi-omics data for novel mixed subgroup identification using machine learning methods
Source: PLoS One. 2023 Oct 19;18(10):e0287176. doi: 10.1371/journal.pone.0287176 (PMC10586677; doi:10.1371/journal.pone.0287176)

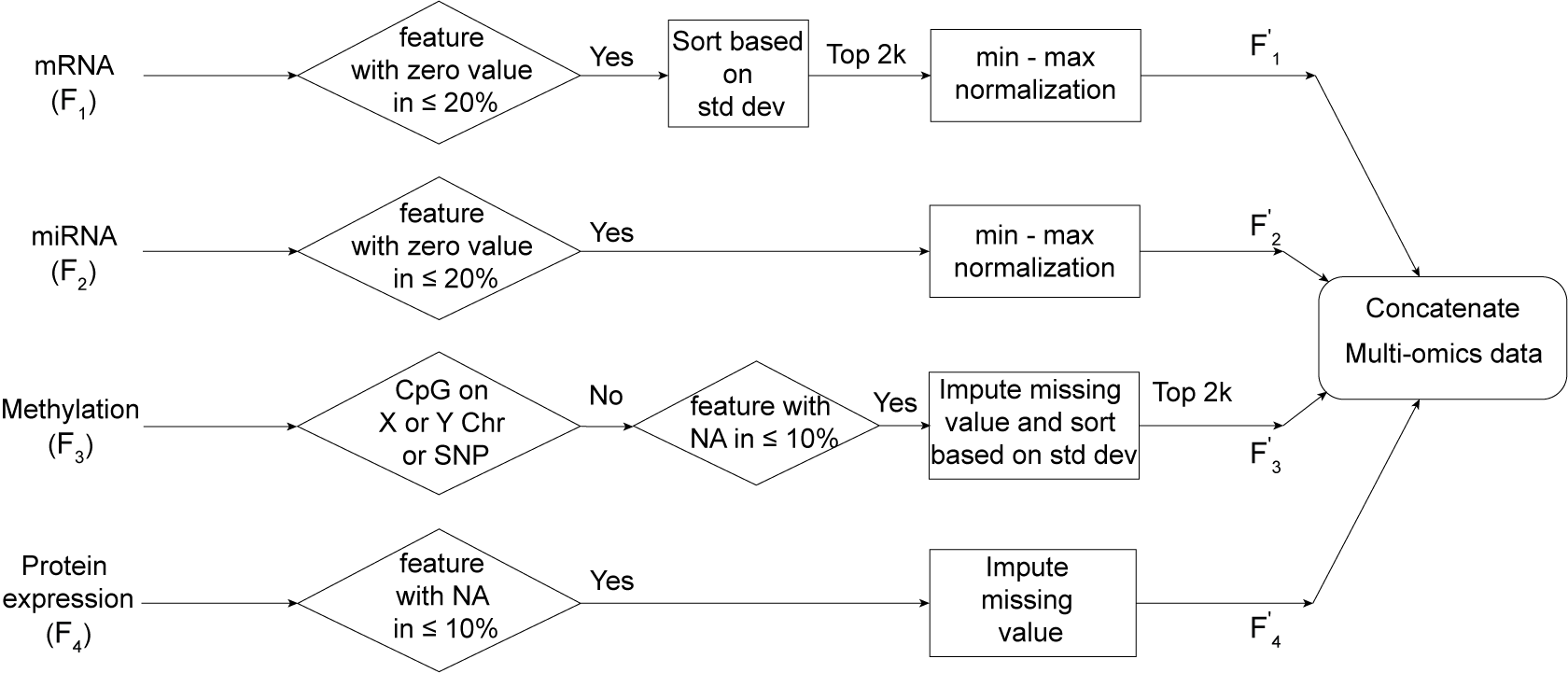

Supplement: S1 Fig — (TIF) [file pone.0287176.s001.tif]

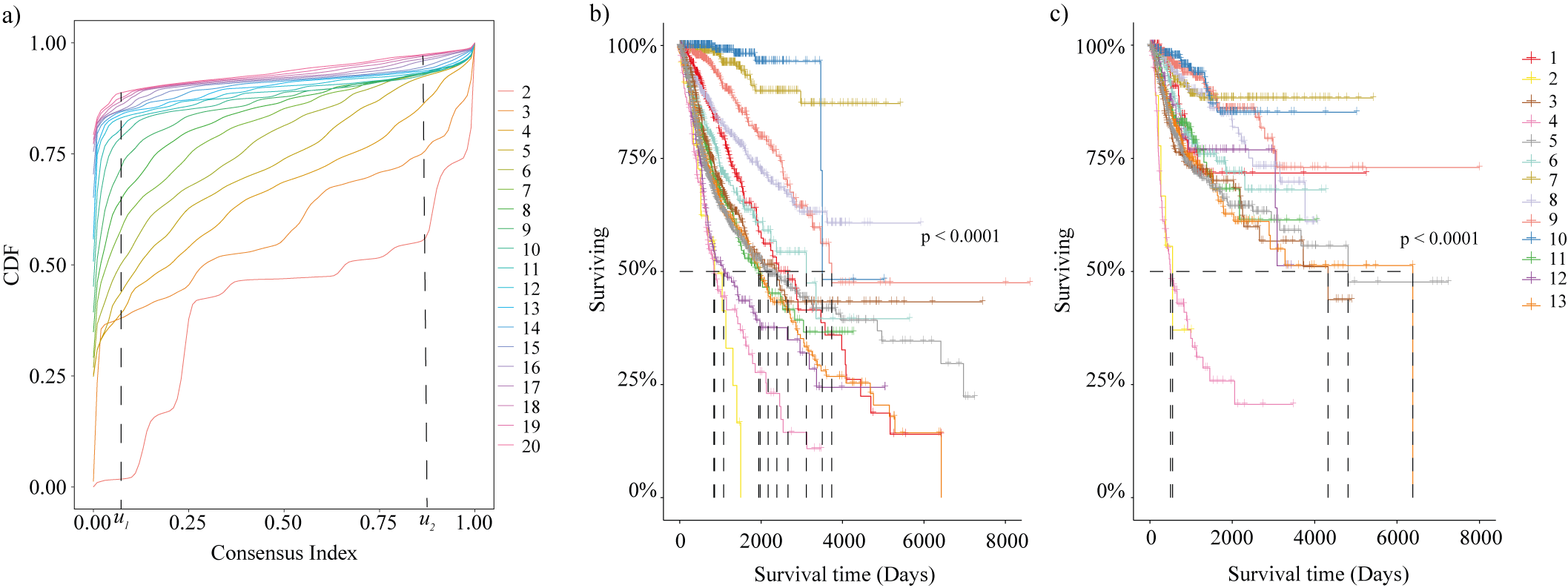

Supplement: S2 Fig — (a) Figures showing the CDF curves for consensus clustering from K = 2 to 20. KM curves for (b) overall survival (OS) and (c) disease-free survival (DFS) in the ML clusters. (TIF) [file pone.0287176.s002.tif]

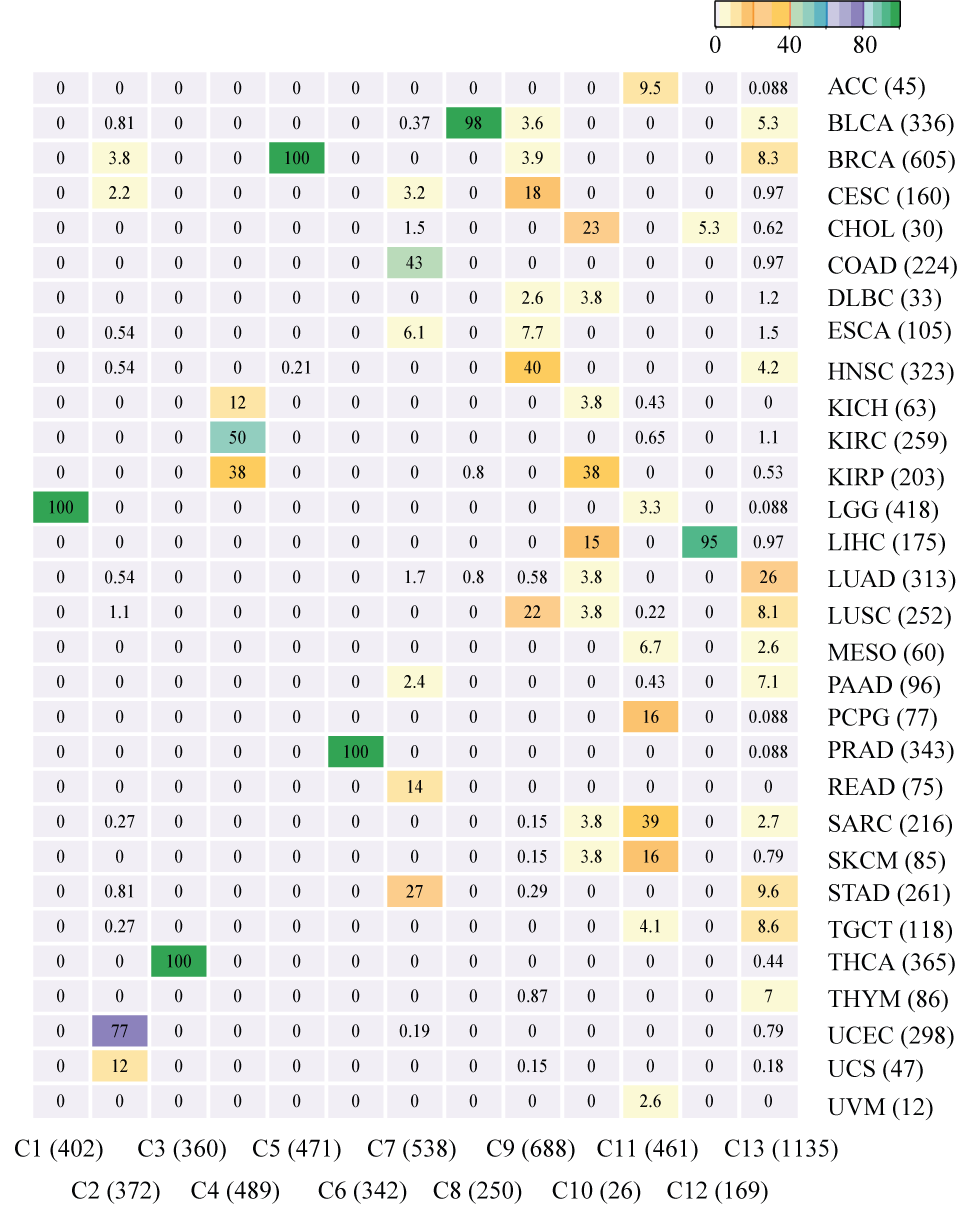

Supplement: S3 Fig — (TIF) [file pone.0287176.s003.tif]

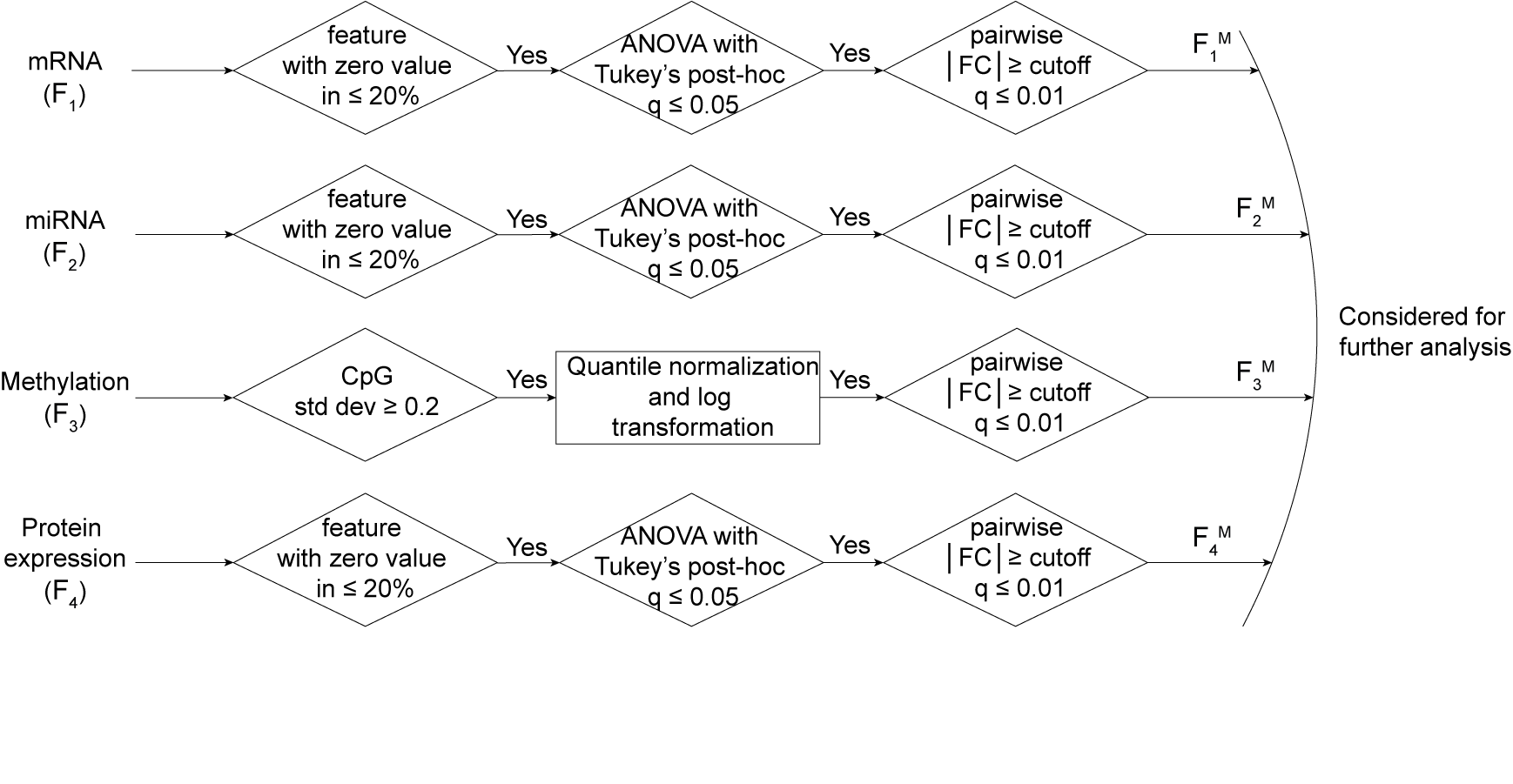

Supplement: S4 Fig — (TIF) [file pone.0287176.s004.tif]

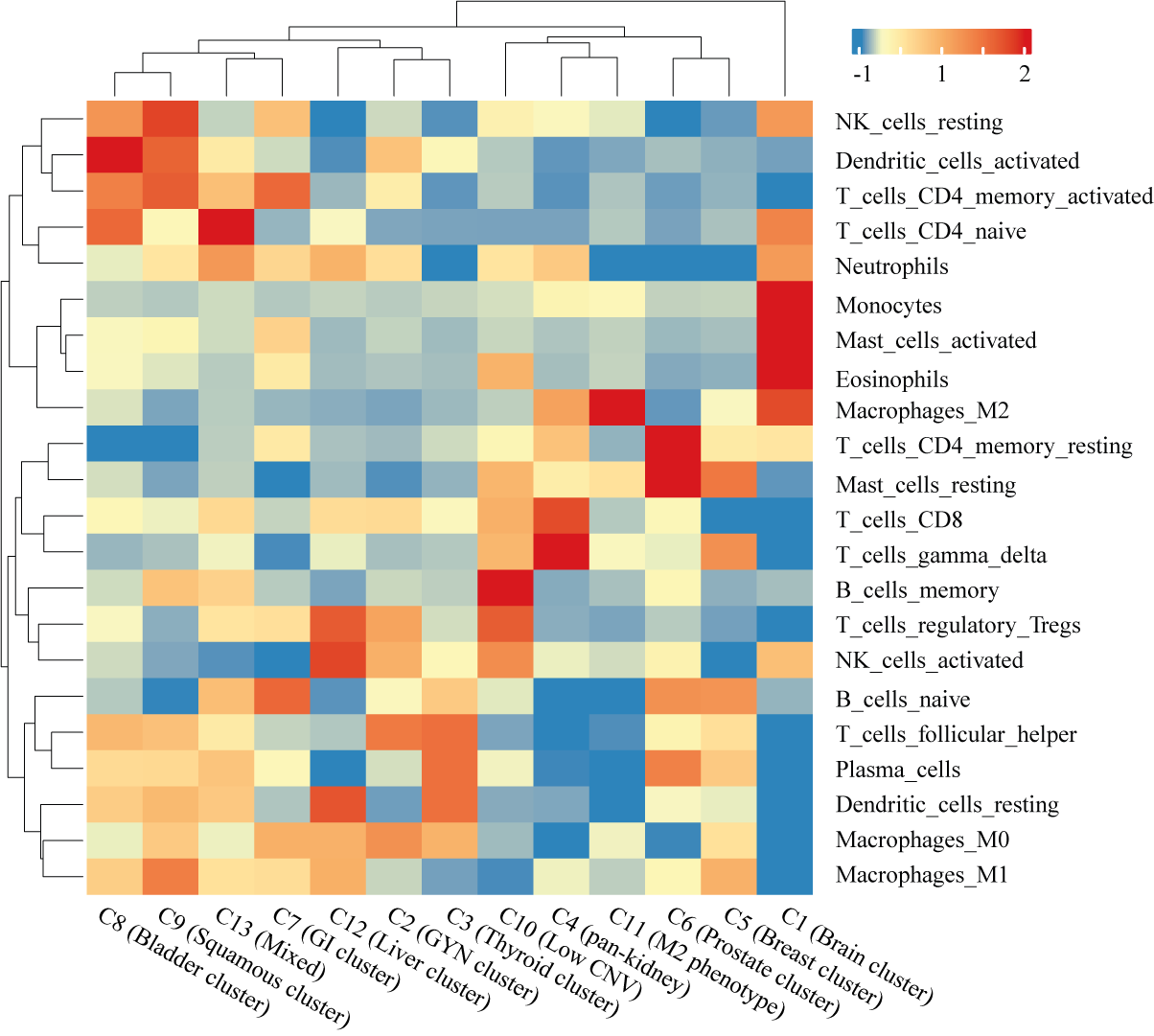

Supplement: S5 Fig — (TIF) [file pone.0287176.s005.tif]

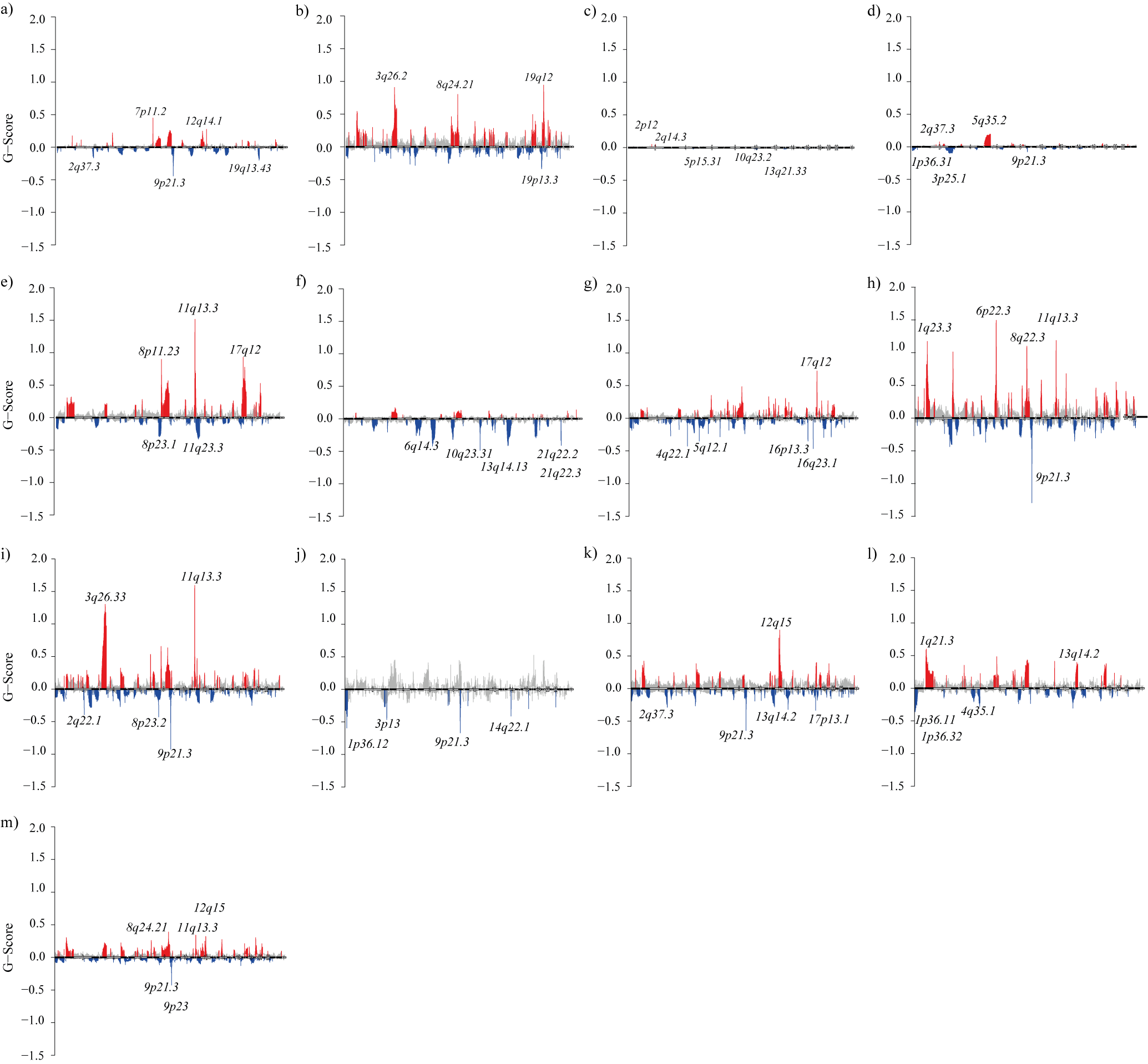

Supplement: S6 Fig — (a) to (m): ML cluster 1 to ML cluster 13. (TIF) [file pone.0287176.s006.tif]

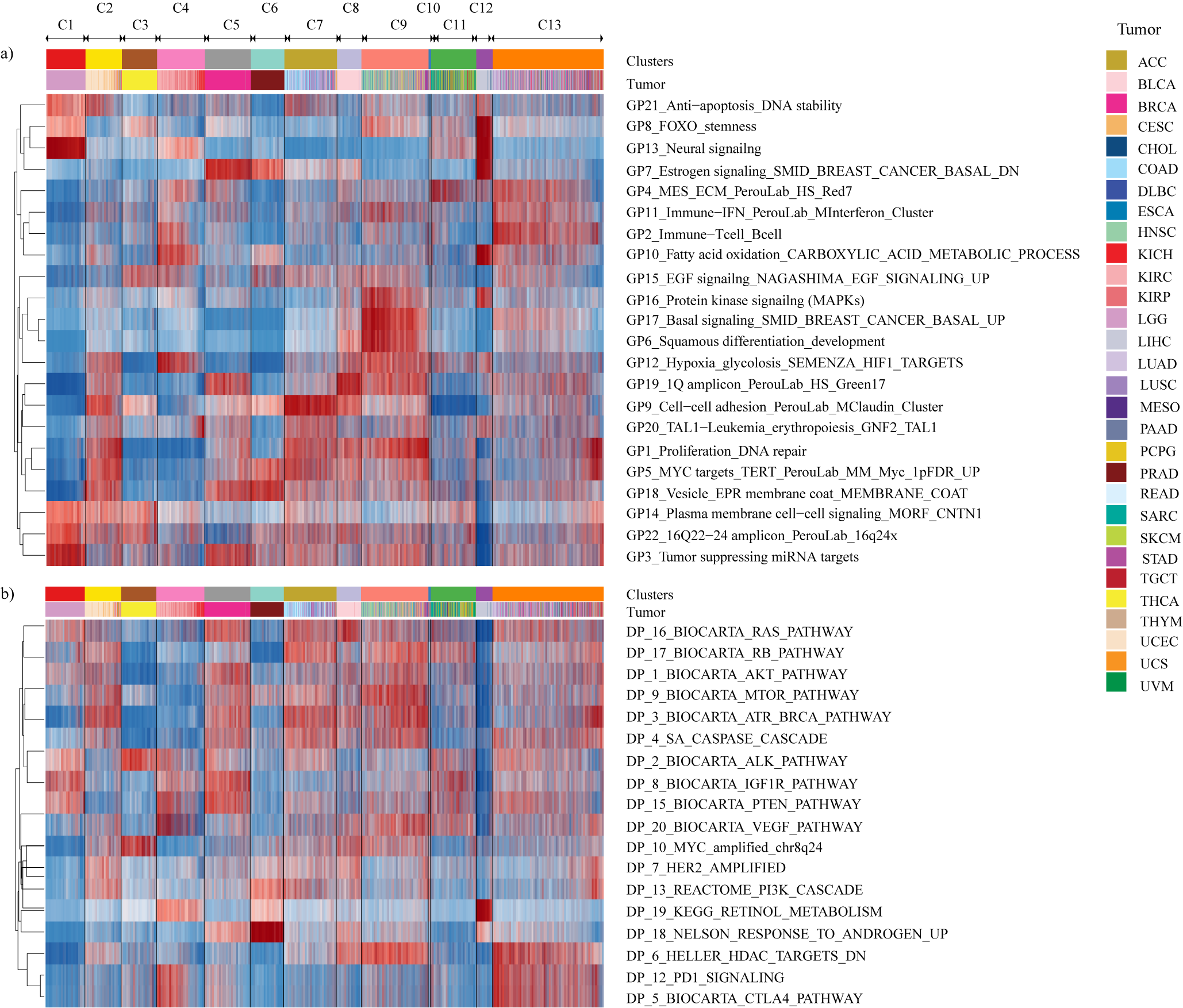

Supplement: S7 Fig — (a) Heatmap showing enrichment of different gene programs in all clusters. (b) Heatmap showing enrichment of different drug programs in all clusters. (TIF) [file pone.0287176.s007.tif]

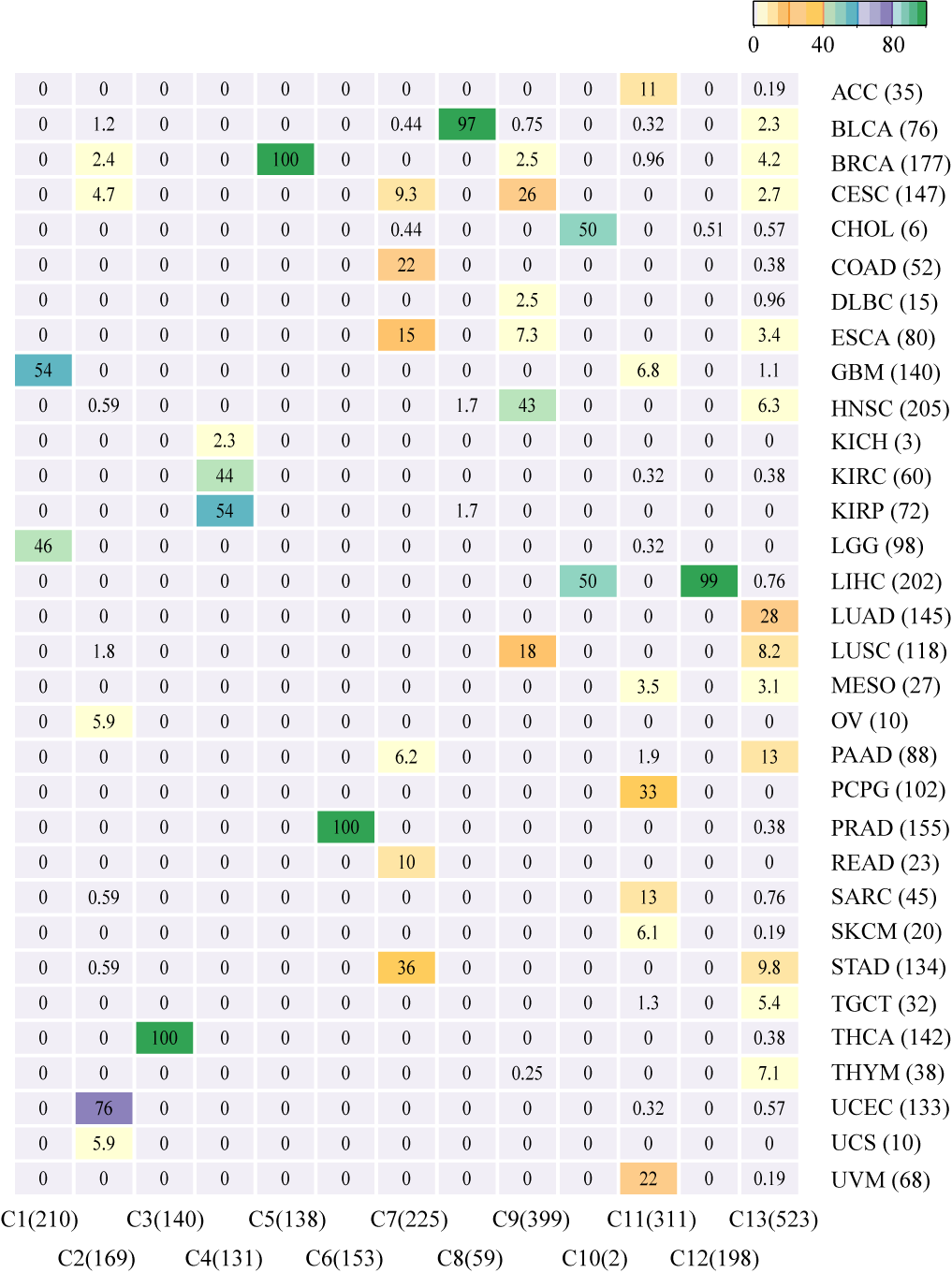

Supplement: S8 Fig — (TIF) [file pone.0287176.s008.tif]

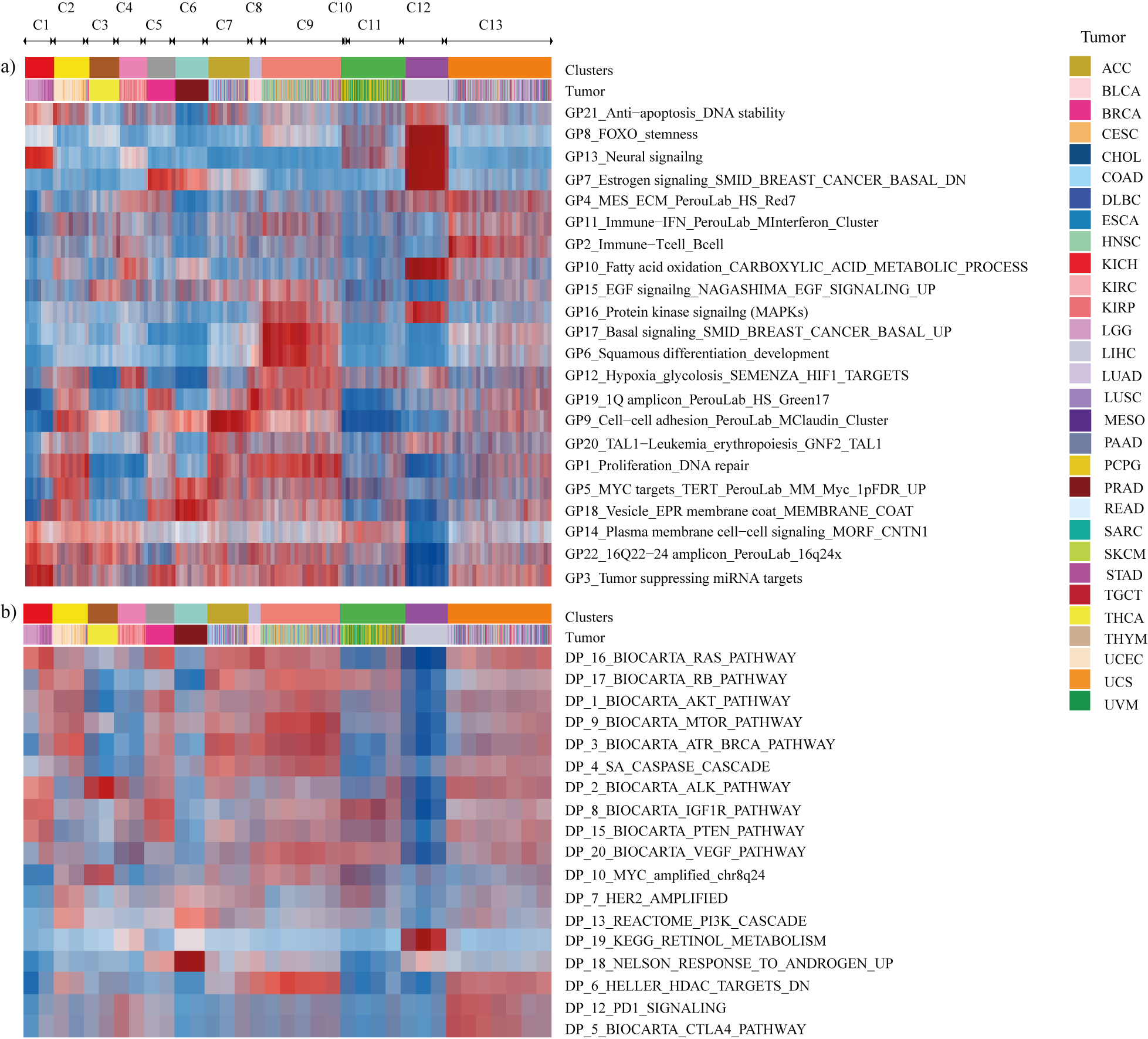

Supplement: S9 Fig — (a) gene and (b) drug programs. (TIF) [file pone.0287176.s009.tif]

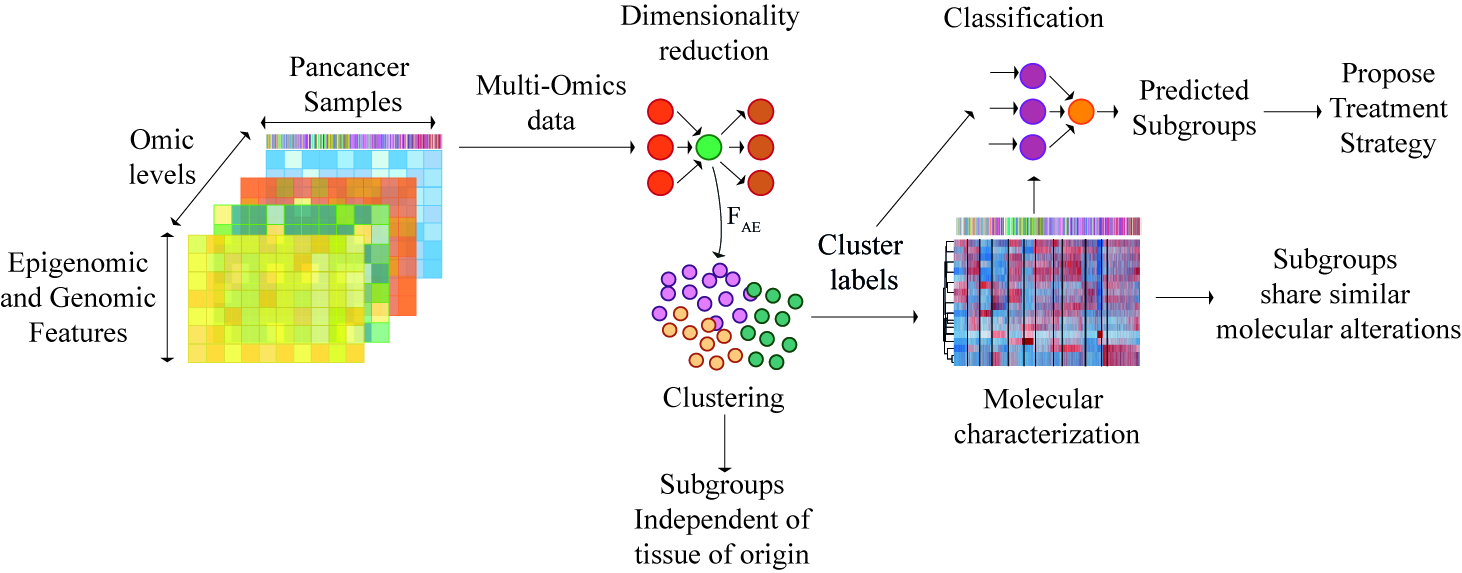

Supplement: S1 Graphical abstract — (TIF) [file pone.0287176.s023.tif]
