# Supplementary material for: Integration of pan-cancer multi-omics data for novel mixed subgroup identification using machine learning methods
Source: PLoS One. 2023 Oct 19;18(10):e0287176. doi: 10.1371/journal.pone.0287176 (PMC10586677; doi:10.1371/journal.pone.0287176)
Supplement: S1 File — (PDF) [file pone.0287176.s022.pdf]

The algorithm used to compute the values of α, β, and γ used to compute the prediction probabilities for the linear decision-level fused classification model.

S1 Algorithm: Calculate α, β, and γ

$\alpha = 0: 0.01: 1$

for α_i in α:

  $\beta = 0 : 0.01 : (1 - \alpha_i)$

  for β_j in β:

    $\gamma = 1 - \alpha_i - \beta_j$

  end for

end for
